# *Sinocorophium hangangense* sp. n. (Crustacea, Amphipoda, Corophiidae), a new species from Korea, with a key to the genus *Sinocorophium*

**DOI:** 10.3897/zookeys.181.3043

**Published:** 2012-04-06

**Authors:** Young-Hyo Kim

**Affiliations:** 1 Canadian Museum of Nature, Research Services, P.O. Box 3443, Station D, Ottawa, Canada K1P 6P4; 2 Department of Life Sciences, Dankook University, Cheonan, Korea 330-714

**Keywords:** Amphipoda, Corophiidae, *Sinocorophium*, new species, Korea, key

## Abstract

A new species of the corophiid gammaridean amphipod belonging to the genus *Sinocorophium* Bousfield & Hoover was collected from the lower reaches of the Han River in Gyeonggi-do, Korea. A relatively large body size and morphology of the uropods 1 and 3 are the major characteristics which serve to distinguish the new species from its congeners. The new species is fully illustrated and extensively compared with related species. A key to the species of (*Sinocorophium* is also provided.

## Introduction

The genus *Sinocorophium* is usually found free-burrowing, in intertidal muddy substrata, from marine to brackish waters. To date, sinocorophiid amphipods are comprised of 10 species (Shen 1955, Bousfield and Hoover 1997) and almost all species reported are endemic to warm temperate and subtropical shallows of the Far East region such as China, Japan, Vietnam and Korea. Only one species, *Sinocorophium alienense* Chapman, 1988 occurs in the northeast Pacific (delta of San Francisco Bay). According to Chapman (1988), *Sinocorophium alienense* was introduced from Vietnam through widespread ballast water traffic during the Vietnam War. The genus *Sinocorophium* is morphologically characterized by having uncoalesced urosomites, uropod 1 laterally inserted, peduncular article 2 of antenna 2 with a large gland cone, palp article 2 of maxilliped elongated and a rounded posteroventral corner of epimeron 3. This genus is divided into two groups, a relatively ancestral and more derived subgroup based on characteristics of antenna 2, gnathopod 1, pereopods 3–4 and uropods (Bousfield and Hoover 1997). The derived subgroup is comprised of 6 species: *Sinocorophium homoceratum* (Yu, 1938), *Sinocorophium monospinum* (Shen, 1955), *Sinocorophium intermedium* (Dang, 1965), *Sinocorophium triangulopedarum* (Hirayama, 1990) from China Sea, *Sinocorophium japonicum* (Hirayama, 1984) from Japan, and *Sinocorophium alienense* (Chapman, 1988) from California. In this paper I add one new Korean species *Sinocorophium hangangense* sp. n., which is placed into the derived subgroup. Hitherto, only one species of the genus, *Sinocorophium sinense* (Zhang, 1974) has been previously recorded in Korea, from intertidal oyster beds (Jung and Kim 2007). A key to the world *Sinocorophium* species is also given.

## Material and methods

**Figure 1.**
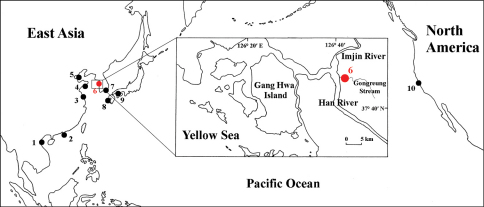
Distribution of world *Sinocorophium* species. **1** *Sinocorophium minutum*, *Sinocorophium intermedium* **2** *Sinocorophium triangulopedarum* **3** *Sinocorophium monospinum* **4** *Sinocorophium sinense* **5** *Sinocorophium heteroceratum, S. homoceratum* **6** *Sinocorophium hangangense* sp. n. **7** *Sinocorophium sinense* **8** *Sinocorophium japonicum*, *Sinocorophium lamellatum* **9** *Sinocorophium sinense* **10** *Sinocorophium alienense*.

Specimens were collected with a hand-net from the mud substratum of Gongreung stream, Paju-si, Korea, where the brackish water region is influenced by the intertidal zone (Fig. 1). The specimens were fixed with 80% ethyl alcohol and dissected in glycerol on Cobb’s aluminum hollow slide. Permanent mounts were made using polyvinyl lactophenol with lignin pink added. Drawings and measurements were performed with the aid of a drawing tube, mounted on an Olympus SZX 12 stereomicroscope and Olympus BX 51 interference contrast compound microscope. The body length was measured from the tip of rostrum to the end of the telson, along the dorsal parabolic line of the body. Type specimens are deposited at the National Institute of Biological Resources (NIBR), Incheon, Korea, Department of Biological Science, Dankook University (DKU), Cheonan, Korea and the Canadian Museum of Nature (CMN), Ottawa, Canada.

## Taxonomy

### Genus Sinocorophium

Bousfield & Hoover, 1997

http://species-id.net/wiki/Sinocorophium

#### Type species.

*Corophium sinensis* Zhang, 1974

#### Diagnosis.

Body cylindrical. Rostrum vestigial to distinct. Antenna 2 strongly pediform, gland cone of peduncular article 2 large, prominent, peduncular article 4 with ventrodistal tooth. Maxilliped, palp article 2 elongate. Gnathopod 1, palm of propodus distinct and transverse, dactylus rather short. Pereopods 3–4, carpus not shortened, slightly shorter than merus. Pleonal epimeron 3 subquadrate or weakly pointed posteroventrally. Urosomites separate. Uropod 1 laterally inserted. Uropod 3 uniramous, ramus linear to subovate. Telson short and subtriangular.

#### Species composition.

*Sinocorophium alienense* (Chapman, 1988), *Sinocorophium hangangense* sp. n., *Sinocorophiumheteroceratum* (Yu, 1938), *Sinocorophium homoceratum* (Yu, 1938), *Sinocorophium intermedium* (Dang, 1965), *Sinocorophium japonicum* (Hirayama, 1984), *Sinocorophium lamellatum* (Hirayama, 1984), *Sinocorophium minutum* (Dang, 1965), *Sinocorophium monospinum* (Shen, 1955), *Sinocorophium sinense* (Zhang, 1974), and *Sinocorophium triangulopedarum* (Hirayama, 1990).

### Sinocorophium hangangense sp. n.

urn:lsid:zoobank.org:act:F79F7494-083C-4E1E-96C7-324B1F712E43

http://species-id.net/wiki/Sinocorophium_hangangense

**Figure 2.**
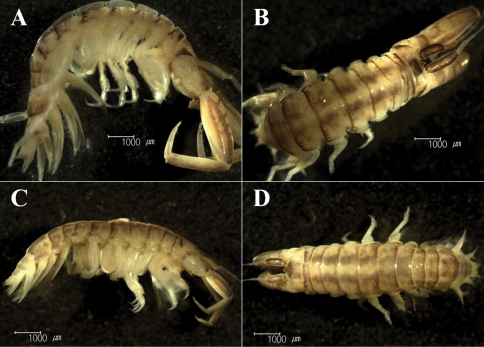
*Sinocorophium hangangense* sp. n., male, 11.5 mm, Gongreung stream, Songchon-ri, Gyoha-eup, Paju-si, Korea. **A** lateral view **B** dorsal view; female, 8.5 mm, Gongreung stream, Songchon-ri, Gyoha-eup, Paju-si, Korea. **C** lateral view **D** dorsal view.

Figs 2 [Fig F3] [Fig F4] 5

#### Korean name:

Han-gang-baem-yeop-sae-u, new

#### Material examined.

Holotype, adult male, 12.2 mm, NIBRIV0000245089, Gongreung stream, Songchon-ri, Gyoha-eup, Paju-si, Gyeonggi-do, Korea, 37°45'10'N, 126°42'20'E, C.M. Lee and Y.H. Kim, 2 November 2002. Paratype, adult female, 11.0 mm, NIBRIV0000245090, same station data; one male, 11.5 mm and one female, 10.5 mm, CMNC 2012-0002, same station data; three females, 8.7–10.2 mm, DKU 201203, same station data.

#### Additional material examined.

2 males, same locality as holotype, C.M. Lee, 30 September 2006; 2 males, 3 females, same locality as holotype, C.M. Lee, 4 November 2006; 5 males, 4 females, same locality as holotype, C.M. Lee, 3 May 2008.

#### Coloration in alcohol.

Body yellowish grey; antennae to urosomites with light brownish reticulate pattern dorsally, especially pereonites with 2 longitudinal rows of light brown lines dorsally (Fig. 2).

#### Description.

**Holotype, adult male**, NIBRIV0000245089.

**Figure 3. F3:**
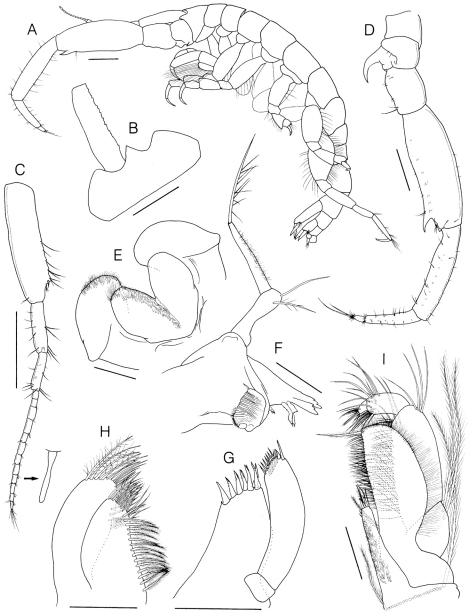
*Sinocorophium hangangense* sp. n., holotype, male, 12.2 mm, Gongreung stream, Songchon-ri, Gyoha-eup, Paju-si, Korea. **A** habitus, lateral **B** head, dorsal **C** antenna 1 **D** antenna 2 **E** lower lip **F** mandible **G** maxilla 1 **H** maxilla 2 **I** maxilliped. Scale bars: 1.0 mm (**A–D**), 0.2 mm (**E–I**).

Body (Fig. 3A) 12.2 mm long, head longer than pereonite 1, rostrum (Fig. 3B) pointed distally, triangular in dorsal view. Eye invisible in alcohol. Cephalic lobe sharply produced. Pereonites 1–2 subequal in length, shorter than pereonite 3. Coxae flat dorsoventrally, except coxa 1, much shallower than pereonites. Urosomites 1–3 separate.

Antenna 1 (Figs. 3B, 3C) weakly setose, subequal in length to head and pereonites 1–4 combined; peduncular article 1 rectangular, distinctly narrowed distally, medial margin irregularly serrated when viewed dorsally, ventrodistal corner with 1 small robust seta and 1 penicillate seta, distal half of ventral margin with 8 setae; length ratio of peduncular articles 1–3 = 1.00 : 0.41 : 0.31; flagellum 13-articulate, shorter than peduncle, several articles bearing club-shaped, small aesthetascs ventrodistally.

Antenna 2 (Figs. 3A, 3D) massive, nearly twice as long as antenna 1; peduncular article 2 with large curved and sharply pointed gland cone; peduncular article 3 longer than wide; peduncular article 4 1.23 × article 5, with a row of tubercles ventromedially and a ventrodistal large tooth; peduncular article 5 rectangular, with a row of tubercles ventromedially; flagellum biarticulate, proximal one with a row of tubercles, 0.56 × peduncular article 5, distal one short, about 0.2 × proximal one, with 2 unequal setae apically.

Lower lip (Fig. 3E) inner lobe subovate, coalescent proximally, rounded apically; mandibular process small and blunt; both lobes covered with patch of pubescence medially.

Left mandible (Fig. 3F) well developed, incisor and lacinia mobilis produced inward, bluntly tridentate; accessory setal row with 2 curved, finely pectinate blades; molar well developed, massive, truncate; palp biarticulate, proximal segment shorter than distal, with 1 simple seta apically and 1 sparse plumose seta subapically, distal segment slender, with pubescence medially and long plumose seta apically.

Maxilla 1 (Fig. 3G) inner plate unknown; outer plate armed with 7 setal-teeth (simple or serrulate) apically; palp biarticulate, proximal segment short, wider than long, distal one extending beyond end of outer lobe, with row of 7 simple setae and 2 unequal robust setae apically, with row of 7 setae subapically.

Maxilla 2 (Fig. 3H) inner plate with longitudinal row of pinnate setae on inner margin, apical margin with 2 rows of simple or pectinate setae; outer plate extending beyond end of inner one, inner distal and apical margins with simple or plumose setae.

Maxilliped (Fig. 3I) inner plate slender and elongate, inner surface covered by pubescence, basal portion with transverse row of about 22 plumose setae, apical margin with 3 unequal robust setae, 1 pinnate and 2 simple setae, respectively; outer plate not reaching distal end of article 2 of palp, inner margin straight, densely setose, with 1 long pinnate seta distally, outer margin pubescent, curved convexly; palp 4-articulate, proximal article with 2 long plumose setae on outer margin, article 2 elongate, more than twice length of proximal one, inner margin densely setose, outer margin with 4 simple setae distally, article 3 subrectangular, with rounded distal corner, surrounded by setae distally, 0.34 × article 2, distal article short, 0.33 × article 3, with apical setae.

**Figure 4. F4:**
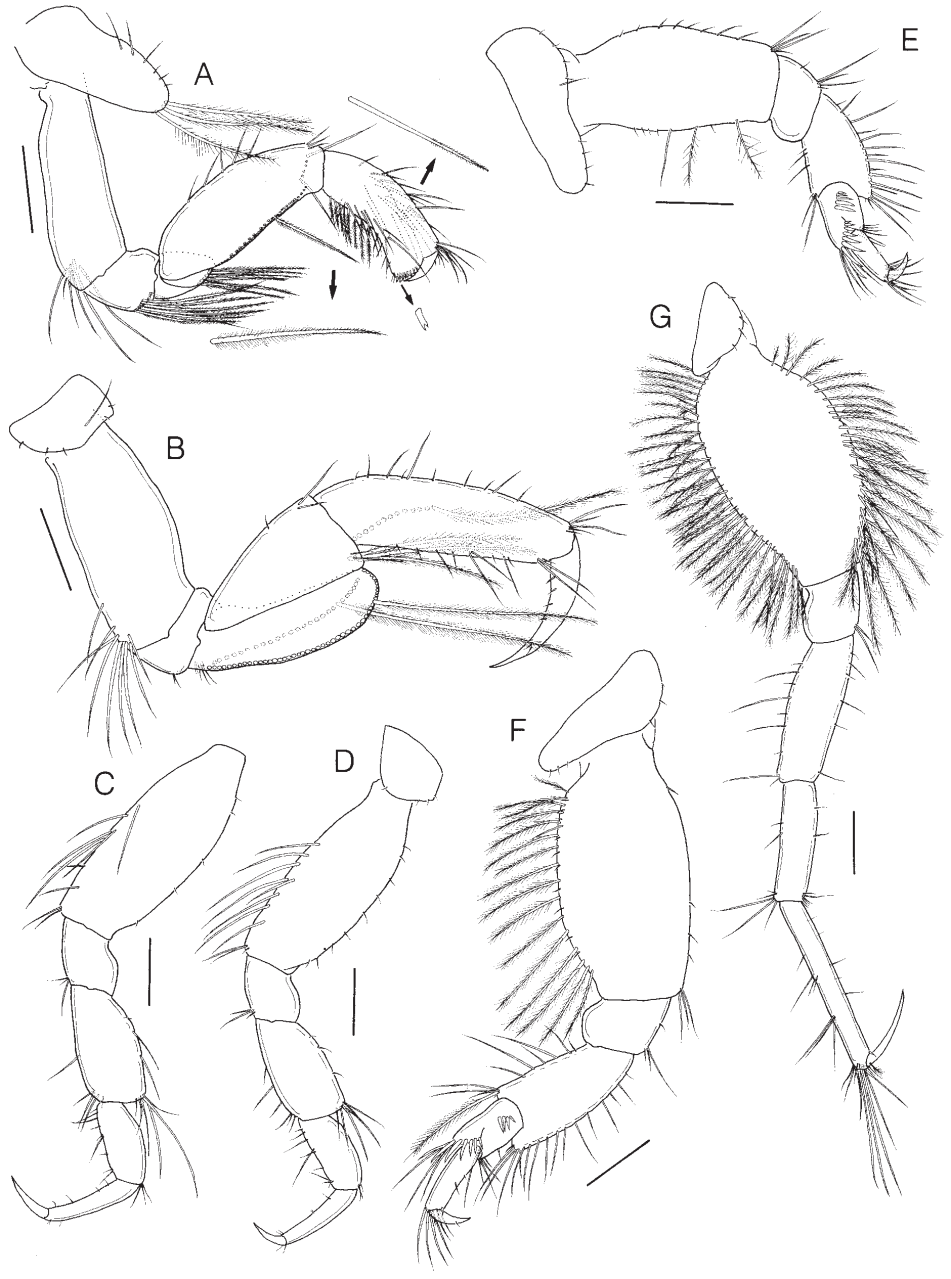
*Sinocorophium hangangense* sp. n., holotype, male, 12.2 mm, Gongreung stream, Songchon-ri, Gyoha-eup, Paju-si, Korea. **A** gnathopod 1 **B** gnathopod 2 **C** pereopod 3 **D** pereopod 4 **E** pereopod 5 **F** pereopod 6 **G** pereopod 7. Scale bars: 0.4 mm (**A–G**).

Gnathopod 1 (Fig. 4A) subchelate; coxa elongate-ovate, much longer than wide, ventral margin rounded with 3 long plumose setae, anterior margin with 6 setules; basis as long as carpus, anterior margin straight, unarmed, posterodistal corner with unequal setae; ischium quadrate, with long pinnate setae ventrodistally; merus short, subtriangular, with long pinnate setae ventrodistally; carpus slightly narrowing distally, anterior margin with 5 simple setae, distomedial corner with transverse row of 6 simple setae, posterior margin with 2 rows of pinnate setae; propodus subrectangular, posterior margin concave, 0.74 × carpus, anterior and medial portions with pectinate setae, posterior margin with pinnate and simple setae, palm transverse, slightly convex, lined with row of bifid spinules; dactylus falcate, almost fitting palm.

Gnathopod 2 (Fig. 4B) simple; coxa small, wider than long; basis subrectangular, posterodistal margin with cluster of simple setae; ischium flat, depressed; merus convexly curved posteriorly, with 2 rows of long pinnate setae along posterior margin and medial portion; carpus isosceles triangle in shape, strongly widening distally, with several pinnate setae posterodistally; propodus weakly narrowing distally, 1.57 × carpus, both margins with unequal simple setae, proximal half of medial portion with oblique row of pinnate setae; dactylus long and falcate, curved concavely, inner margin with row of setules.

Pereopod 3 (Fig. 4C) coxa small, wider than long; basis weakly expanded medially, anterior margin with 5 setules, distal half of posterior margin with simple setae; merus slightly widening distally, 1.24 × propodus; carpus rather elongated, 0.69 × merus; dactylus simple, 0.52 × propodus.

Pereopod 4 (Fig. 4D) similar to pereopod 3.

Pereopod 5 (Fig. 4E) coxa depressed, much wider than long, slightly concave midventrally, narrowing distally; basis slightly widened medially, anteromarginally with row of setules, posteromarginally with sparse setae; merus widening distally, both margins with unequal simple setae; carpus with 2 oblique rows of 5 proximal and 8 distal robust setae respectively, subequal in length to propodus; dactylus short.

Pereopod 6 (Fig. 4F) similar to pereopod 5, but about 1.3 × longer; basis more greatly expanded posteriorly, with a row of setules and plumose setae; merus slightly widening distally, both margins with simple setae, posterodistally with 1 plumose seta; propodus slender, rectangular, 1.13 × carpus; dactylus about 0.5 × propodus.

Pereopod 7 (Fig. 4G) elongate, much longer than either pereopod 5 or 6; coxa small, ventral margin convexly rounded, with 4 setules; basis elongate-ovate, moderately expanded anteroposteriorly, densely setose along both margins with long plumose setae; ischium to propodus linear and rectangular; length ratio of articles 2–7 = 1.00 : 0.31 : 0.67 : 0.48 : 0.75 : 0.36.

**Figure 5.**
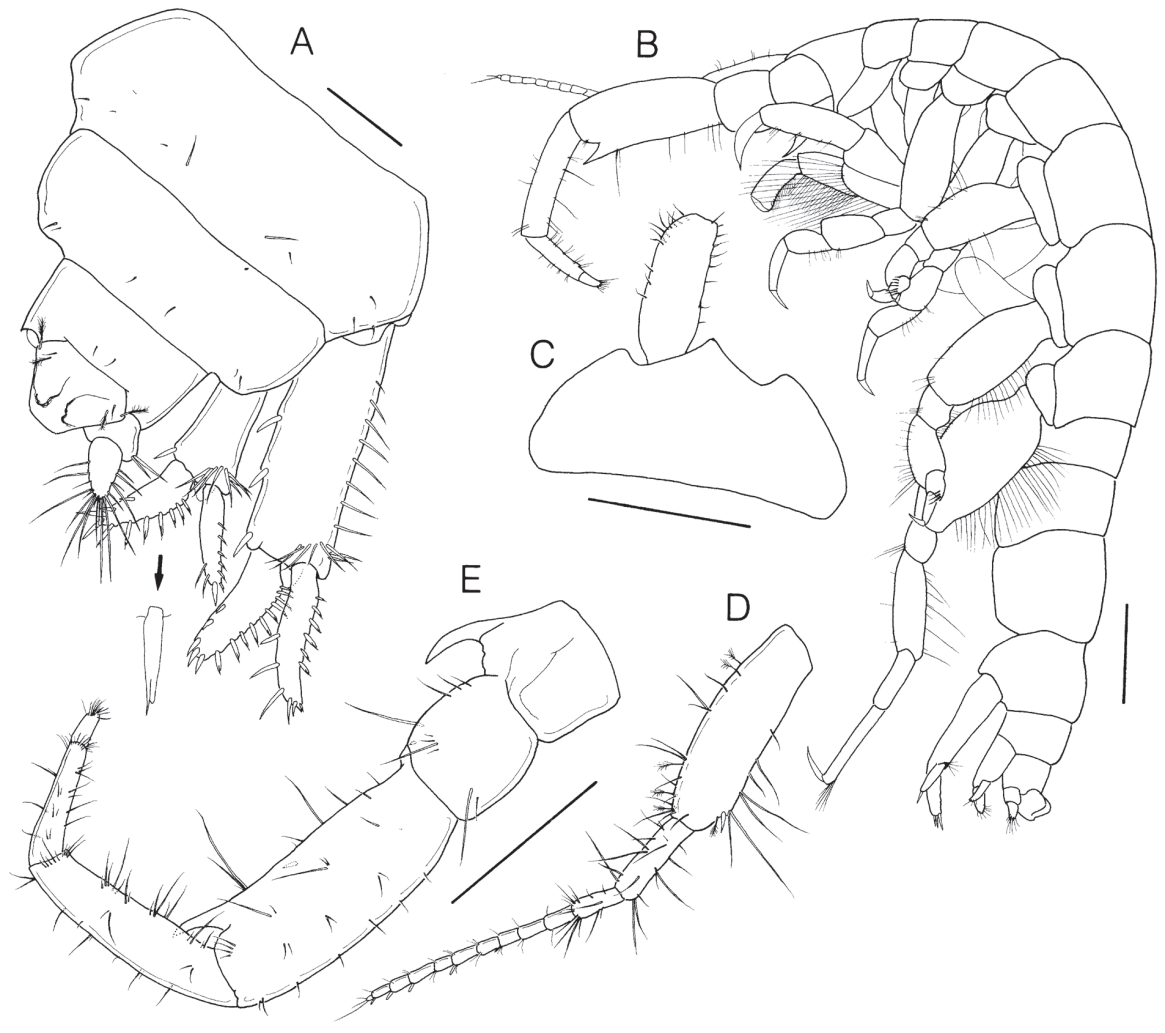
*Sinocorophium hangangense* sp. n., holotype, male, 12.2 mm, Gongreung stream, Songchon-ri, Gyoha-eup, Paju-si, Korea. **A** urosomites, uropods and telson, dorsal; paratype, female, 11.0 mm, Gongreung stream, Songchon-ri, Gyoha-eup, Paju-si, Korea. **B** habitus, lateral **C** head, dorsal **D** antenna 1 **E** antenna 2. Scale bars: 1.0 mm (**B–E**), 0.4 mm (**A**).

Urosomites 1–3 (Fig. 5A) separate; urosomite 1 longest, widest in middle when viewed dorsally, urosomite 2 longer than 3, nearly rectangular, posterodistal margins rounded in dorsal view; uropods 1–3 arising laterally.

Uropod 1 (Fig. 5A) slightly extending beyond end of uropod 2; peduncle rectangular, 1.84 × outer ramus, ventrodistal process present, triangular, blunt, lateral margin with row of simple setae, medial one with 3 robust setae; outer ramus slightly longer than inner, lateral margin with 6 robust setae, medial one with 5 robust setae, including 2 subdistal robust setae; inner ramus slightly curved medially.

Uropod 2 (Fig. 5A) peduncle slightly longer than rami, with triangular ventrodistal process, apicodistal robust setae and cluster of setae; rami subequal in length, with robust setae marginally.

Uropod 3 (Fig. 5A) uniramous, peduncle short, broader than long, 0.78 × ramus; ramus subelliptical, narrowing distally, margins with unequal simple setae, with apical setae.

Telson (Fig. 5A) fleshy, thickened, grooved centrally, subtriangular, with truncate corners, broadest in middle, dorsolaterally with 2 penicillate setae and 1 setule.

**Paratype, female** (sexually dimorphic characters), 11.0 mm, NIBRIV0000245090.

Body (Fig. 5B) similar to male including antenna 2, but rostrum (Fig. 5C) weaker; antenna 1 (Fig. 5D) peduncular article 1 without medial serrations, article 3 rather short; antenna 2 (Fig. 5E) subsimilar to that of male, but less robust and shorter, peduncular article 4 with 1 ventromedial robust seta.

#### Remarks.

Bousfield and Hoover (1997) divided *Sinocorophium* into two groups, a relatively ancestral and a more derived subgroup through a numerical taxonomic analysis. According to this classification, the new species belongs to the derived subgroup. Some characteristics which relate to this group include non sexual dimorphic antenna 2, convex palm of the gnathopod 1, short carpi of pereopods 3–4 and short ramus of the uropod 3 (Table 1). However, the new species shows more similarity with the ancestral subgroup in having an elongate-ovate ramus of uropod 3 which is longer than the peduncle. The new species is distinguished from its congeners by having a row of slender setae and not robust setae on the lateral margin of the peduncle of uropod 1. These are unique morphological systematic statuses among the more derived subgroup which is sexually subsimilar in antenna 2. Ecologically the new species inhabits lower stream regions with lowered salinity, while related congeners except *Sinocorophium intermedium* and *Sinocorophium minutum* inNorth Vietnam are mainly abundant in shallow subtidal and intertidal mudflats. Among the derived subgroup species, the new species is more similar to *Sinocorophium alienense* of the northeast Pacificthan to the geographically closer sinocorophiid amphipods in the Far East. These two species have several characteristics in common: 1) prominent ventromedial teeth row on peduncular articles 4–5 and first flagellum of male antenna 2; 2) inner ramus of uropod 1 curved medially and shorter than outer one; 3) antenna 2, posterodistal tooth of peduncular article 4 unidentate; 4) basis of pereopod 5 bearing posteromarginal setae. However, the new species is distinguished from *Sinocorophium alienense* (different characters of *Sinocorophium alienense* in brackets) by the combination of the following features: 1) peduncular article 1 of male antenna 1 with ventral simple setae (with pinnate setae); 2) peduncular article 1 of antenna 1 about 2.3 × article 2 (peduncular article 1 subequal in length to article 2); 3) peduncular article 1 of antenna 1 with serrated medial margin (with smooth margin); 4) each lobe of telson with mid-lateral 1 simple and 2 penicillate setae, respectively (unornamented).

**Table 1.** Morphological characters of *Sinocorophium hangangense* sp. n. and related species with sexually subsimilar antenna 2.<br/>

Character and distribution	Species						
	*Sinocorophium alienense* (♂)	*Sinocorophium homoceratum* (♂)	*Sinocorophium intermedium* (♂)	*Sinocorophium japonicum* (♀)	*Sinocorophium monospinum* (♀)	*Sinocorophium triangulopedarum* (♂)	*Sinocorophium hangangense* sp. n. (♂)
Body length	6.5 mm	10–12 mm	8.0 mm	6.0 mm	4.5 mm	5.5 mm	12.2 mm
Rostrum	subequal to cephalic lobe	subequal to cephalic lobe	subequal to cephalic lobe	vestigial	longer than cephalic lobe	subequal to cephalic lobe	subequal to cephalic lobe
Cephalic lobe	subacute	rounded	rounded	weak	rounded	subacute	subacute
Antenna 1, peduncular article 1 ventral margin	pinnate setae	8 teeth	?	setae	setae	simple setae	simple setae
Antenna 1, length of peduncular article 1 to article 2	=	>>	>	>	>	>	>>
Antenna 1, flagellum, # of articles	13–15	16–18	17	12	14	17?	13
Antenna 2, ventrodistal process	1	2	1	1	1	1	1
Mandible, length of palp articles1 & 2	proximal < distal	subequal	proximal < distal	subequal	subequal	proximal > distal	proximal < distal
Maxilliped, length of outer plate	less than end of palp article 2	exceeding end of palp article 2	?	less than end of palp article 2	reaching end of palp article 2	less than end of palp article 2	less than end of palp article 2
Pereopod 5, posterior margin of basis	setose	setose	?	not setose	?	not setose	not setose
Uropod 1, lateral margin of peduncle	robust setae	robust setae	?	robust setae	robust setae	robust setae	slender setae
Uropod 1, length of rami	inner < outer	inner << outer	inner << outer	subequal	inner << outer	inner < outer	inner < outer
Uropod 3, shape of ramus	ovate	clavate	ovate	ovate	ovate	semi-circular	elongate-ovate
Uropod 3, length of ramus	< peduncle	= peduncle	= peduncle	= peduncle	= peduncle	< peduncle	> peduncle
Habitat	intertidal	tide-pool	brackish	intertidal	coastal	mangrove marsh	brackish
Distribution	San Francisco Bay, California (Chapman 1988)	Tangku, Hopei, Yellow Sea (Yu 1938)	Thanh hoa, Vietam, Gulf of Tongking (Dang 1965)	Tomioka Bay, Kyushu, Japan (Hirayama 1984)	Fenghsien, Kiangsu, East China Sea (Shen 1955)	Mai po, Hong Kong, South China Sea (Hirayama 1990)	Paju-si, Korea (present study)

#### Etymology.

Named for the type locality, Gongreung stream, which is a small tributary on the lower reaches of the Han River in Gyeonggi-do, Korea. The Korean word “Gang” means river.

#### Habitat.

The new species is found in brackish water regions, but it may inhabit intertidal regions, are downstream of the type locality.

#### Distribution.

Songchon-ri, Gyoha-eup, Paju-si, Gyeonggi-do, Korea.

##### Key to the species of *Sinocorophium*

**Table:** 

1	Antenna 2 sexually dimorphic; gnathopod 1, palm steeply oblique to transverse; pereopods 3–4, merus subequal to carpus	2
–	Antenna 2 sexually subsimilar; gnathopod 1, palm gently convex; pereopods 3–4, merus longer than carpus	5
2	Body medium sized, more than 5 mm; mandibular palp, terminal article short, much shorter than proximal article	3
–	Body small sized, less than 5 mm; mandibular palp, terminal article normal, subequal to proximal article	4
3	Antenna 2(♀), peduncular article 4 with 2 ventral robust setae; gnathopod 2(♂), basis with dentiform process anterodistally	*Sinocorophium sinense* (Zhang, 1974)
–	Antenna 2(♀), peduncular article 4 without ventral robust seta; gnathopod 2(♂), basis straight, without dentiform process anterodistally	*Sinocorophium heteroceratum* (Yu, 1938)
4	Rostrum minute, vestigial	*Sinocorophium lamellatum* (Hirayama, 1984)
–	Rostrum rather prominent	*Sinocorophium minutum* (Dang, 1965)
5	Rostrum short, vestigial; uropod 1, rami subequal in length	*Sinocorophium japonicum* (Hirayama, 1984)
–	Rostrum elongate; uropod 1, inner ramus shorter than outer	6
6	Antenna 2, peduncular article 4 with 2 ventrodistal large teeth	*Sinocorophium homoceratum* (Yu, 1938)
–	Antenna 2, peduncular article 4 with 1 ventrodistal large tooth	7
7	Antenna 2, peduncular article 4 stubby, more than 0.5 × as wide as long	*Sinocorophium intermedium* (Dang, 1965)
–	Antenna 2, peduncular article 4 elongate, less than 0.5 × as wide as long	8
8	Body large sized, more than 10 mm; uropod 1, lateral margin of peduncle with setae; uropod 3, ramus longer than peduncle	*Sinocorophium hangangense* sp. n.
–	Body small sized, less than 7 mm; uropod 1, lateral margin of peduncle with robust setae; uropod 3, ramus subequal or shorter than peduncle	9
9	Antenna 1, peduncular article 1 subequal in length to article 2	*Sinocorophium alienense* (Chapman, 1988)
–	Antenna 1, peduncular article 1 longer than article 2	10
10	Uropod 3, ramus semi-circular, shorter than peduncle	*Sinocorophium triangulopedarum* (Hirayama, 1990)
–	Uropod 3, ramus ovate, subequal in length to peduncle	*Sinocorophium monospinum* (Shen, 1955)

## Supplementary Material

XML Treatment for Sinocorophium

XML Treatment for Sinocorophium hangangense
